# The protective effect of persistent trigeminal artery in patients with ischemic stroke

**DOI:** 10.1186/s12883-019-1374-7

**Published:** 2019-07-11

**Authors:** Yicheng Xu, Yong Kong, Yahui Xu, Peifu Wang

**Affiliations:** Department of Neurology, Aerospace Center Hospital, Yuquan Road, NO.15, Haidian District, Beijing, 100049 People’s Republic of China

**Keywords:** Persistent trigeminal artery (PTA), Cerebrovascular disorders, Stroke, Anastomosis, Risk

## Abstract

**Background:**

Almost all case reports related to persistent trigeminal artery indicated that the existence of persistent trigeminal artery may increase the risk of ischemic stroke. However our case demonstrated that the persistent trigeminal artery may also play a protective role in preventing severe ischemic stroke by functioning as collateral circulation.

**Case presentation:**

We reported a patient with left internal carotid artery occlusion with persistent trigeminal artery manifesting only as a minor acute ischemia stroke exhibiting acute onset of dizziness and difficulty in walking. Brain MRI showed two small areas of restricted diffusion on diffusion-weighted imaging in the left hemisphere. The digital subtraction angiography showed his left middle cerebral artery and bilateral anterior cerebral artery were supplied by the basilar artery via a persistent trigeminal artery. Furthermore, CT perfusion showed no remarkable difference between the two hemispheres.

**Conclusions:**

Persistent trigeminal artery may have a protective role in the setting of an acquired occlusion of homolateral internal carotid artery. Therefore, it is important to fully assess the presence of the persistent trigeminal artery in acute ischemic stroke.

## Background

Persistent trigeminal artery (PTA) is one of the most common types of the carotid- vertebrobasilar anastomoses occurred incidentally in adults, with reported incidence of 0.1 to 0.6% of all cerebral angiograms [1]. Due to its anatomical proximity to the trigeminal nerve, there have been some case reports on its association with trigeminal neuralgia. Similarly, there are also some reports on the associations between the PTA with intracranial aneurysms and carvernous sinus fistulas [2, 3]. Moreover it is rarely reported in the setting of acute ischemia stroke, especially with the internal carotid artery (ICA) occlusion. Meanwhile, Almost all of these case reports related to stroke indicated that it may increase the risk of ischemic stroke [4–6]. However, its protective effect in the setting of ischemic stroke was rarely reported. Herein, we reported a case of left ICA occlusion with PTA manifesting only as a minor acute ischemia stroke. And its role in cerebrovascular disease has also been discussed.

## Case presentation

A 64-year-old man with a 13-year history of coronary heart disease and 7-year history of hypertension was admitted to our hospital for acute onset dizziness and unsteady gait for one week. He reported acute onset of dizziness and difficulty in walking, and these symptoms would become worsened when he got up from the bed or sofa. On admission, his blood pressure was 135/85 mmHg and heart rate was 66 beats/min; and neurological examination revealed nystagmus on horizontal gaze. His pupillary reflexes and extraocular movements were intact, no limb weakness and sensory deficits were found; and bilateral finger--to-nose and heel-to-shin tests were normal. Additionally, bilateral Babinski signs were absent. However, Romberg sign was impossible to evaluate as the patient could not be able to cooperate with further examination.

**Fig. 1:**
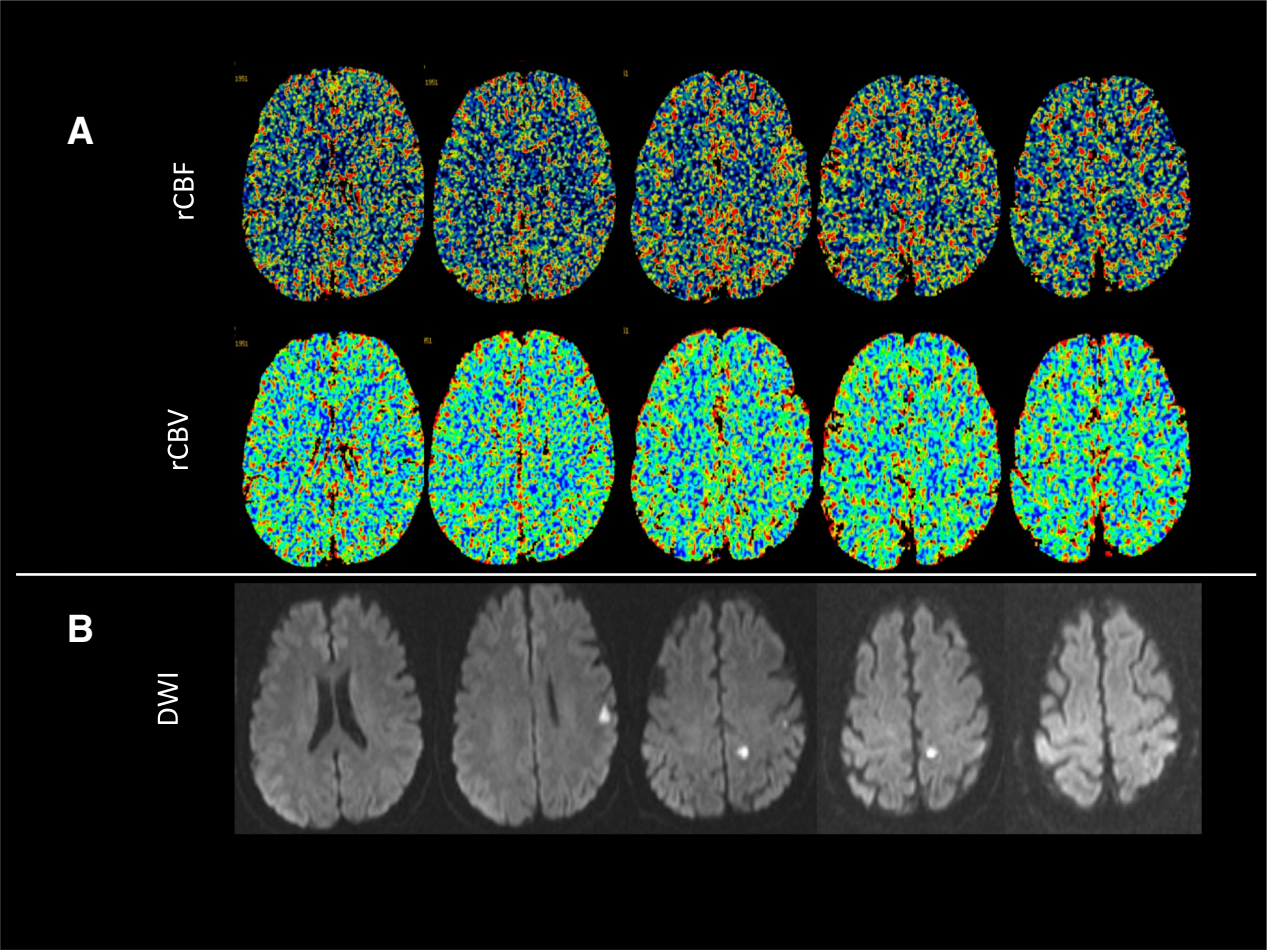
CT perfusion and MR imaging (**a**) CT perfusion: CBF and CBV show no remarkable difference between the bilateral. Hemispheres. (**b**) DWI: acute infarcts in the left MCA territory. DWI = diffusion –weighted imaging; MCA = middle cerebral artery; rCBF = regional cerebral blood flow; rCBV = regional cerebral volume

**Fig. 2:**
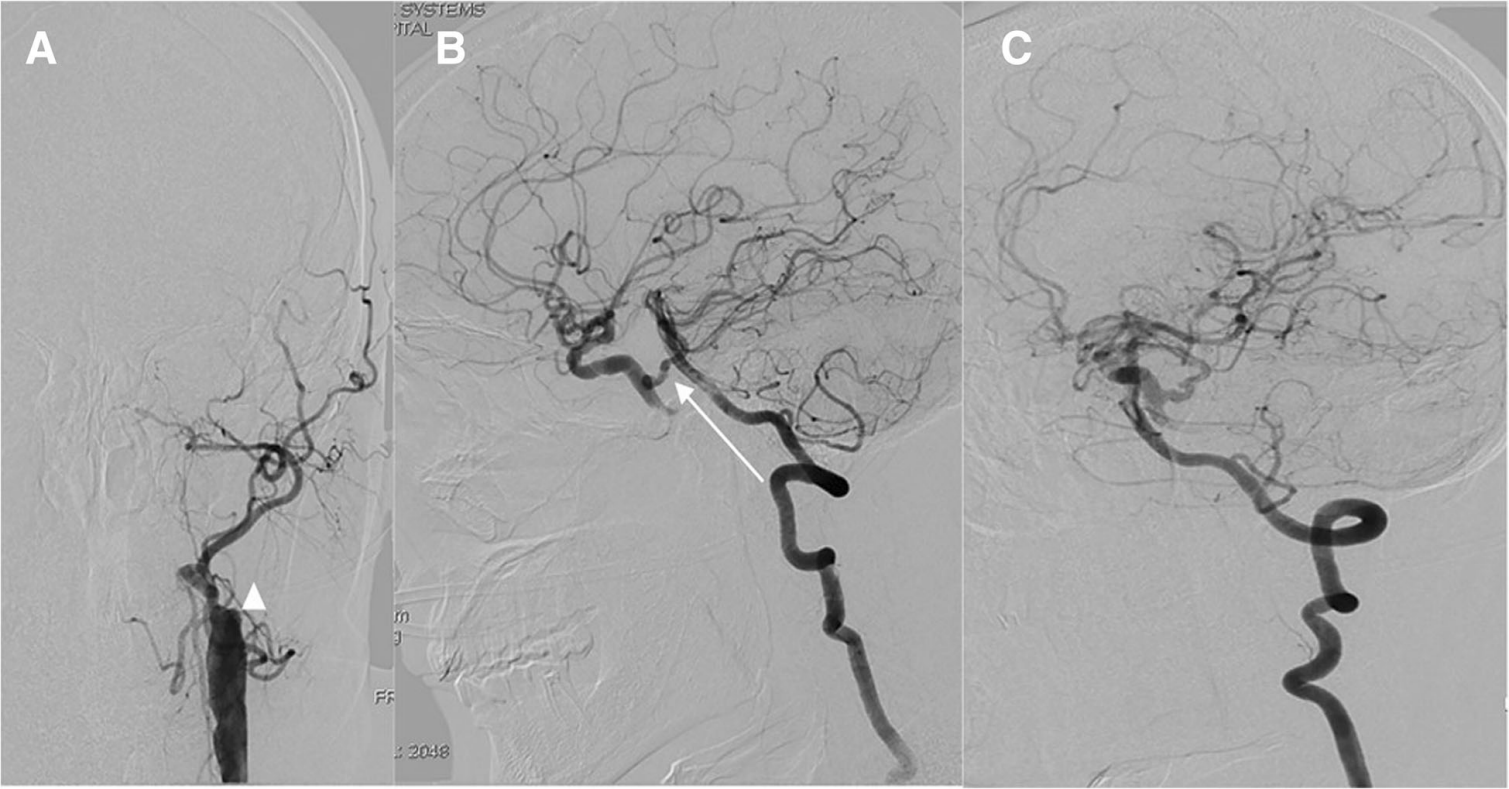
The trigeminal artery in DSA. Triangular arrowhead points to the occlusion of the left internal carotid artery (**a**). White arrow points to the trigeminal artery (**b**). DSA demonstrates the reversal of the blood flow in the trigeminal artery from the BA to the left ICA to supply blood to the left MCA and the bilateral ACA (**c**). DSA = digital subtraction angiogram; ICA = internal carotid artery; BA = basilar artery; MCA = middle cerebral artery; ACA = anterior cerebral artery

Similarly, laboratory tests were within normal limits. Brain MRI performed 10 h after admission demonstrated two areas of restricted diffusion on diffusion-weighted imaging (DWI) in the left hemisphere (Fig. 1b). However, no acute infarction was seen in the posterior cerebral artery territories. Considering the location of infarction area could not well explain his clinical symptoms, a digital subtraction angiography (DSA) was done which revealed that the left ICA was occluded (Fig. 2a). Meanwhile, it showed that the left middle cerebral artery and bilateral anterior cerebral artery were supplied by the basilar artery via a PTA (Fig. 2b + c). Based on that we presumed that in the setting of acute left ICA occlusion, a cerebral blood flow steal phenomena occurred from posterior circulation to anterior circulation which caused the patient to have acute onset of dizziness and difficulty in walking. We also speculated that the PTA may play a protective role in preventing the patient from having severe anterior circulation ischemic stroke. Furthermore, CT perfusion showed no remarkable difference between the two hemispheres (Fig. 1a), which was consistent with the above conclusion. Therefore, the patient was given 100 mg aspirin and 20 mg atorvastatin daily and discharged one week later. During two years follow-up, the patient had no recurrence of stroke.

## Discussion and conclusions

The role of PTA in ischemic stroke has not been fully understood. The vast majority of case reports on the role of PTA in the setting of ischemic cerebrovascular disease supported the viewpoint that the existence of PTA may increase the risk of ischemic stroke [4–6]. This mechanism has been well explained by the hypothesis that PTA may play a role as an embolic pathway to the posterior circulation from the ICA [4]. Some other rare instances had also been reported such as PTA thrombosis resulting from the extensive of thrombosis in the occlusive carotid dissection attributed to brainstem infarction [7]. Similarly, the compression of pons from the PTA also could cause brainstem transient ischemia stroke [8]. These two aforementioned cases are also in line with the above viewpoint that, existence of PTA may increase the risk of ischemic stroke.

This case report has some strength. Firstly, our case indicated that the PTA may also play a protective role in preventing severe ischemic stroke by functioning as collateral circulation. This was consistent with the finding that the PTA has a collateral support value in patients who underwent the balloon occlusion test of the ICA [9]. Secondly, to the best of our knowledge, this was the first case in a real patient that demonstrated the protective role of PTA in the setting of acute occlusive cerebrovascular disease by using the combination of the cerebral angiogram and CT perfusion. In most instances, the flow direction of patients with PTA was from the carotid to the basilar artery, which well explained the reason why patient with ICA stenosis and PTA may develop ischemic stroke of the posterior circulation [10]. However, in accordance with the case of Lochner and colleagues [11], the cerebral angiogram of our case showed that the flow direction of the PTA was from the basilar artery to the ICA territory. Moreover, since there was no remarkable difference between the two hemispheres, the CT perfusion further proved the protective role of PTA. So it can be assumed that the PTA may be crucial to the patients in the presence of ICA occlusion or severe stenosis.

In conclusion, PTA may have a protective role in the setting of an acquired occlusion of homolateral ICA. Therefore, it is important to fully assess the presence of the PTA in acute ischemia stroke.

## Data Availability

Not applicable.

## Abbreviations

DSA: Digital subtraction angiography
DWI: Diffusion-weighted imaging
ICA: Internal carotid artery
PTA: Persistent trigeminal artery
